# En Route towards European Clinical Breakpoints for Veterinary Antimicrobial Susceptibility Testing: A Position Paper Explaining the VetCAST Approach

**DOI:** 10.3389/fmicb.2017.02344

**Published:** 2017-12-15

**Authors:** Pierre-Louis Toutain, Alain Bousquet-Mélou, Peter Damborg, Aude A. Ferran, Dik Mevius, Ludovic Pelligand, Kees T. Veldman, Peter Lees

**Affiliations:** ^1^UMR 1331 Toxalim, INRA, ENVT, Toulouse, France; ^2^The Royal Veterinary College, University of London, London, United Kingdom; ^3^Department of Veterinary and Animal Sciences, University of Copenhagen, Frederiksberg, Denmark; ^4^Wageningen Bioveterinary Research, Lelystad, Netherlands; ^5^National Reference Laboratory on Antimicrobial Resistance in Animals, Lelystad, Netherlands

**Keywords:** Antimicrobial Susceptibility Testing, VetCAST, breakpoints, veterinary, antimicrobials

## Abstract

VetCAST is the EUCAST sub-committee for Veterinary Antimicrobial Susceptibility Testing. Its remit is to define clinical breakpoints (CBPs) for antimicrobial drugs (AMDs) used in veterinary medicine in Europe. This position paper outlines the procedures and reviews scientific options to solve challenges for the determination of specific CBPs for animal species, drug substances and disease conditions. VetCAST will adopt EUCAST approaches: the initial step will be data assessment; then procedures for decisions on the CBP; and finally the release of recommendations for CBP implementation. The principal challenges anticipated by VetCAST are those associated with the differing modalities of AMD administration, including mass medication, specific long-acting product formulations or local administration. Specific challenges comprise mastitis treatment in dairy cattle, the range of species and within species breed considerations and several other variable factors not relevant to human medicine. Each CBP will be based on consideration of: (i) an epidemiological cut-off value (ECOFF) – the highest MIC that defines the upper end of the wild-type MIC distribution; (ii) a PK/PD breakpoint obtained from pre-clinical pharmacokinetic data [this PK/PD break-point is the highest possible MIC for which a given percentage of animals in the target population achieves a critical value for the selected PK/PD index (*f*AUC/MIC or *f*T > MIC)] and (iii) when possible, a clinical cut-off, that is the relationship between MIC and clinical cure. For the latter, VetCAST acknowledges the paucity of such data in veterinary medicine. When a CBP cannot be established, VetCAST will recommend use of ECOFF as surrogate. For decision steps, VetCAST will follow EUCAST procedures involving transparency, consensus and independence. VetCAST will ensure freely available dissemination of information, concerning standards, guidelines, ECOFF, PK/PD breakpoints, CBPs and other relevant information for AST implementation. Finally, after establishing a CBP, VetCAST will promulgate expert comments and/or recommendations associated with CBPs to facilitate their sound implementation in a clinical setting.

## Introduction

There is increasing concern on the public health and animal welfare consequences of antimicrobial resistance (AMR) in bacteria from animal sources. The appropriate use of antimicrobial drugs (AMDs) in veterinary medicine is one of the key areas of European Union (EU) policy objectives to combat AMR. Various initiatives have been taken by many national, European and international bodies to promote prudent use of AMDs (European Platform for Responsible Use of Medicines in Animals, 2008; European Commission, 2011; Federation of Veterinarians of Europe, 2012; European Food Safety Authority, 2014; European Medicines Agency, 2015; World Health Organisation, 2015; World Organisation for Animal Health, 2016).

The importance of bacterial diagnostics and Antimicrobial Susceptibility Testing (AST) as the basis for a rational choice of an antimicrobial to treat an infection has been advocated in numerous international and national guidelines and publications (Federation of Veterinarians of Europe, 2012; European Medicines Agency, 2015; World Health Organisation, 2015; World Organisation for Animal Health, 2016), and by the European Committee on AST – EUCAST^1^. Therefore, there is an urgent need to achieve, in the EU, reliable interpretive criteria for AST, which are harmonized and evidence-based. This will ensure optimized and controlled AMD prescribing and use. Currently, only one body, the Clinical Laboratory Standards Institute (CLSI), an internationally recognized non-government organization, with its veterinary sub-committee VAST, has pioneered AST for veterinary medicine. As science is universal, several procedures described in this paper are also inevitably covered by CLSI/VAST documents, procedures and standards. The CLSI methods are described in a series of commercial documents (VET01, VET02, ..). These are not freely available in the public domain, but are available to purchase by CLSI customers at https://clsi.org/standards/products/veterinary-medicine/documents/. In contrast, all VetCAST documents will be freely available as required by general EUCAST policy.

In 2015, VetCAST (Veterinary Committee on AST) was established as a subcommittee of EUCAST (European Committee on AST). VetCAST aims to advise on all aspects of AST for bacterial pathogens of animal origin and animal bacteria with zoonotic potential. An important consideration is standardization of methodology for AST; this is essential to ensure reproducibility of data between laboratories and therefore the valid use of these data to estimate the prevalence of resistance (Franklin et al., 2001). Equally important is the application of inappropriate interpretative criteria to report AST results. If such criteria are inappropriate the test will be of limited or no value for the prescriber, even when the laboratory methodology is standardized and reliable. This is the case when no domestic animal species-specific veterinary interpretative criteria, i.e., Clinical Breakpoints (CBPs), are available.

Comprehensive reviews on setting CBPs for human medicine have been published (Turnidge and Paterson, 2007; Dalhoff et al., 2009; Mouton, 2014), and other reviews have considered their application in veterinary medicine (Constable and Morin, 2002; Apley, 2003; Bywater et al., 2006; Schwarz et al., 2008; Lubbers and Turnidge, 2015). The CLSI/VAST documents, notably the VET01 and VET02 documents cover this topic (Clinical and Laboratory Standards Institute, 2008, 2013). The objective of this manuscript is to describe the procedures used by VetCAST to define CBPs; these procedures will be within the format adopted by EUCAST (see EUCAST SOP 1.2, 2016), but with emphasis on considerations appropriate to veterinary medicine. This procedure incorporates the EUCAST science-based, transparent system for definition of CBPs.

## General Considerations

Antimicrobial Susceptibility Testing is considered as one of the most important factors governing the selection of antimicrobials for clinical veterinary use (De Briyne et al., 2013). The primary objective of AST in animal healthcare is selection of the most appropriate antibiotic for the welfare interests of the animal to be treated. A second important objective of AST is to ensure good veterinary treatment practices, which take into account public health hazards. A third objective is the provision of good epidemiological AST surveillance data. In this respect, AST was shown to be the most sensitive tool for detecting the emergence of new resistance mechanisms (Mather et al., 2016).

In veterinary medicine, as in human medicine, it is commonly accepted that AST data predict the clinical outcome of AMD treatment. In human medicine, however, it has been reported that AST may sometimes fail to provide an accurate prediction of clinical outcome (Doern and Brecher, 2011). Likewise, in veterinary medicine, AST results may not always provide an accurate prediction of clinical outcome. An example of poor predictive value is AST for topical (intra-mammary) AMD administration in mastitis therapy in cattle (Constable and Morin, 2003; Barlow, 2011). One objective of VetCAST is to provide evidence for the predictive value of AST, in terms of both animal and public health.

Many factors account for the fact that AST and the Minimum Inhibitory Concentration (MIC), determined *in vitro* using standardized methodology, cannot reflect all aspects of complex *in vivo* clinical circumstances. These factors were recently reviewed by Martinez et al. (2013). The most important concern for AST arises when no animal species- and infection-specific veterinary CBPs are available; this may lead to the use of inappropriate drugs or doses, potentially resulting in treatment failure and, unintentionally, selection of antimicrobial resistance. Therefore, the first remit of VetCAST is to develop and implement animal species specific CBPs to ensure the provision of scientifically driven and clinically relevant information to antimicrobial stewardship programs (see **Table 1** for the definition of the main terms, abbreviation and acronyms used in this paper).

**Table 1:** Definition of the main terms, abbreviation and acronyms used in this paper.

AMD	Antimicrobial drug
AST	Antimicrobial Susceptibility Testing
AUC	Area under plasma concentration-time curve
AUC/MIC	A PK/PD index; defined as the area under the concentration–time curve at steady-state over 24 h unless otherwise stated.
CART analysis	Classification and Regression Tree Analysis
CBP	Clinical Breakpoint; the values of MIC (mg/L) selected by an *ad hoc* committee to be used by testing laboratories to qualitatively report the results of AST as Susceptible (S), Intermediate (I), or Resistant (R). Not to be confused with cut-offs.
CLSI	The Clinical and Laboratory Standards Institute
CLSI/VAST	Sub-committee of CLSI dealing with the susceptibility testing of veterinary pathogens and determination of veterinary CBP
*C*_max_/MIC	A PK/PD index; the peak plasma concentration divided by MIC
CO	A threshold value for MICs to separate two entities or targets
CO_CL_	Term not used by EUCAST; for CLSI, Clinical cut-off is the MIC value for which probability-of-cure can be achieved with a routine dosage regimen in a given percentage of animals
EARS-Net	European Antimicrobial Resistance Surveillance Network
ECDC	European Center for Disease prevention and Control
ECOFF	Epidemiological (bacteriological) cut-off
EFSA	European Food Safety Authority
EMA	European Medicines Agency
ESCMID	European Society of Clinical Microbiology and Infectious Diseases
EU	European Union
EUCAST	European Committee on Antimicrobial Susceptibility Testing
FAO	Food and Agriculture Organization (part of UN)
FDA	Food and Drug Administration
MCS	Monte Carlo simulation; Monte Carlo methods are a class of computational algorithms that use repeated random sampling to obtain numerical results. In the context of AMDs, MCS is a tool for determining the probability of achieving a specific PK/PD index value defined as a PTA
MBC	Minimum Bactericidal Concentration (mg/L)
MIC	Minimum Inhibitory Concentration (mg/L)
OIE	World Organization for Animal Health (retained OIE as its historical acronym)
PK	Pharmacokinetics; drug disposition
PK/PD breakpoint	Pharmacokinetic/Pharmacodynamic; the MIC value for which a targeted PK/PD index value can be achieved with a routine dosage regimen in a given percentage of animals (usually 90%)
Prediction interval	In statistical inference, specifically predictive inference, a prediction interval is an estimate of an interval in which future observations will fall, with a certain probability, given what has already been observed.
PTA	Probability of Target Achievement/Attainment (synonym of TAR). In Monte Carlo simulations, the probability that at least a specific value of a pharmacodynamic index (e.g., 30% *f*T > MIC; *f*AUC/MIC of 100) is achieved at a certain (minimum inhibitory) concentration.
T > MIC	A PK/PD index; the cumulative percentage of a 24 h period that the drug concentration exceeds the MIC under steady-state pharmacokinetic conditions unless otherwise stated
VetCAST	Veterinary Committee for Antimicrobial Susceptibility Testing
VICH	Veterinary International Conference on Harmonization; VICH is a trilateral (EU–Japan–United States) program aimed at harmonizing technical requirements for veterinary product registration
WHO	World Health Organization

## The Requirement for and Challenges of Establishing Species-Specific, Substance-Specific and Disease-Specific Clinical Breakpoints in Veterinary Medicine

Clinical breakpoints are MIC values (expressed in mg/L), or their surrogates such as zone diameters used by diagnostic laboratories to categorize results of AST as Susceptible (S), Intermediate (I), or Resistant (R). In veterinary diagnostics, CBPs should be defined for each animal species, and for the relevant bacterial target pathogens in each animal species. Several specific veterinary features should be considered when setting CBPs in veterinary medicine. For each animal species, AMDs can be administered by various routes, including parenteral administration (most commonly intramuscular or subcutaneous routes) using formulations with short, intermediate or long durations of action. Formulations for oral administration range from single dose products to products for administration in feed, including medicated feed, milk replacer or drinking water. For these products and especially for the oral route, bioavailability can be very variable, because absorption rate and extent depend on both individual and group animal behaviors. The consequence is large inter-individual differences in pharmacokinetic profiles, which affect markedly the systemic exposure to AMDs and hence therapeutic outcome. Some modalities of AMD administration are specific to veterinary medicine; an important example is intra-mammary administration in dairy cattle. For this route, not only is there no equivalent human CBP, there is no conceptual framework on how to develop a CBP, based on milk concentration in the udder after local intra-mammary administration. All these factors must be taken into consideration when defining CBPs.

A further challenge is posed by within-species differences in drug disposition (Toutain et al., 2010) and response, e.g., pre-ruminant vs. ruminant calves vs. adult ruminants (Martinez and Modric, 2010; Modric and Martinez, 2011). In addition, domestic animal species have evolved into unique breeds with distinguishable characteristics, deriving from genetic selection; this is particularly apparent for dogs but also applies to poultry breeds and other species. When feasible, VetCAST will take into account the pharmacokinetic differences arising from intra-species genetic and other variations. The consequence may be that several CBPs will be defined for an AMD for a given animal species.

The practice of selecting a single AMD to represent all agents in the same pharmacological class, and hence proposing pharmacological class CBPs, will need careful consideration. For individual drugs within classes, both potency and pharmacokinetics may vary widely and this in turn influences the selected dosage regimen and CBP.

## Establishing Clinical Breakpoints by VetCAST

Based on the EUCAST SOP 1.2 (EUCAST SOP 1.2, 2016), VetCAST will establish a formal and transparent approach for the development and determination of CBPs. The rationale underlying this approach is that the selection of a CBP by VetCAST may impact the possible use, misuse or overuse of some drugs.

**FIGURE 1:**
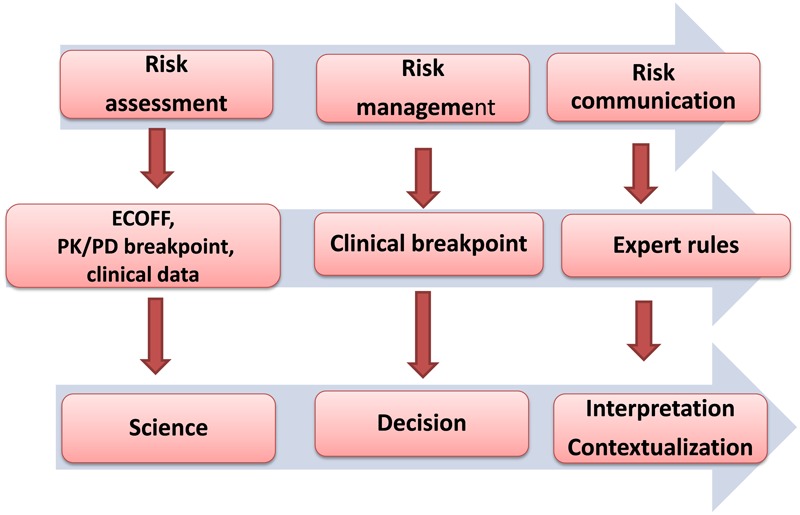
The successive steps in the process for establishing and implementing Clinical Breakpoints (CBPs) by VetCAST will follow three steps. The scientific assessment is a scientifically based process comprising determination of two critical MICs associated to ECOFF and PK/PD breakpoint and of clinically collected surrogates (MIC, AUC_24h_/MIC…) able to predict clinical outcomes. The second step is decision (provisional for review or final for implementation) of a CBP based upon the different pieces of information determined at the previous step. This second step requires independence from all stakeholders. The final step involves communication, and includes the interactive exchange of information on standards, expert comments, guidelines, SOPs, ECOFF, PK/PD breakpoint, the CBP and any matter relating to susceptibility testing between VetCAST and its stakeholders.

The process for establishing CBPs by VetCAST will involve three principal stages: a first scientific step involving assessment of available data; second, a formal procedure for decision taking on the CBP; and finally the recommendations for implementation and use of the CBP (**Figure 1**).

**FIGURE 2:**
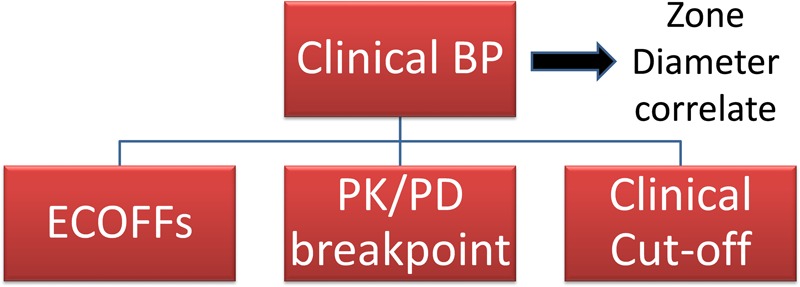
Clinical breakpoints (CBP) are the values of the MIC (mg/L) selected by an *ad hoc* committee to be used by testing laboratories to qualitatively report the results of AST as Susceptible (S), Intermediate (I), or Resistant (R). CBPs are determined by taking into account the ECOFF, the PK/PD breakpoint and the clinical cut-off when available. CBP is established also by taking into account any aspects (scientific or not) that should be considered to ensure harmonization between countries.

*The scientific assessment* to establish a new CBP comprises direct or indirect determination of two or three critical MICs. These can be viewed operationally as MIC cut-off values needed to assist the selection of the CBP (**Figure 2**): (i) an epidemiological cut-off value (ECOFF), described by EUCAST as the *ECOFF* (ii) a MIC to define a PK/PD cut-off, named by EUCAST as the *PK/PD breakpoint*, and (iii) specifically in veterinary medicine a MIC cut-off related to clinical outcomes, the ‘clinical cut-off value.’ The clinical cut-off is not currently described or used by EUCAST in human medicine. The comparative richness of the data generated in human PK/PD and clinical studies obviates the need for such a cut-off. However, it is assessed indirectly when setting CBPs for human pathogens because the value of the PK/PD index target that is determined in clinical studies depends on the MIC collected in individual patients. In veterinary medicine, such data are rather scarce or non-existent, and this creates a need to examine MIC vs. clinical outcome data directly. VetCAST will need to explore some innovative approaches to integrate clinical data, including the clinical cut-off, into the conceptual framework of PK/PD vs. clinical outcome relationship. The CBP is the final MIC value determined by considering all three cut-offs together.

The scientific assessment of the data file required to establish a CBP will involve input from all stakeholders, but especially the veterinary pharmaceutical industry, which could provide raw data with or without a preliminary analysis (e.g., a population pharmacokinetic analysis to derive a PK/PD breakpoint). VetCAST will issue a guideline on methods and standards for collecting, archiving, handling and analyzing pharmacokinetic data, and general guidelines for handling MIC distribution data and determining ECOFFs will be published soon by VetCAST on the EUCAST website. These two guidelines will guarantee the quality of collected data, and a full transparency of procedures in VetCAST’s determination of the three critical MIC cut-offs.

After completion of the scientific assessment, the next stage will be that of CBP selection. The CBP will be determined by taking into account the previously established ECOFFs, PK/PD breakpoints and clinical cut-offs.

This second step requires independence from all stakeholders (public and private), and VetCAST members will decide CBPs by consensus as done by EUCAST. When determined, CBPs will be released as recommended MICs (mg/L), and a publically available rationale document will be issued to explain and justify the numerical values selected. The values of the ECOFFs, PK/PD breakpoints and clinical cut-offs will also be available publicly.

The final step involves the public release of information (except that protected by confidentiality agreements) and issuing of expert rules, as currently undertaken by EUCAST (Leclercq et al., 2013).

For VetCAST, as currently is the case for EUCAST, the veterinary pharmaceutical industry will be a partner and will have an active consultative role, but will not have a financial role or participate in decision-making, as is the case for the CLSI/VAST sub-committee.

## Reporting Test Results

It is well established that a clinically relevant microbiology report should constitute an integral part of any infectious disease management program (Cunney and Smyth, 2000; Guardabassi and Prescott, 2015). To assist clinical microbiologists in preparing their report, VetCAST will provide *ad hoc* guidance documents for interpretation of ASTs. This will ensure the adequate and contextually correct interpretation of AST results in the light of the animal’s local or regional circumstances.

## Epidemiological Cut-Off Values (ECOFF)

**FIGURE 3:**
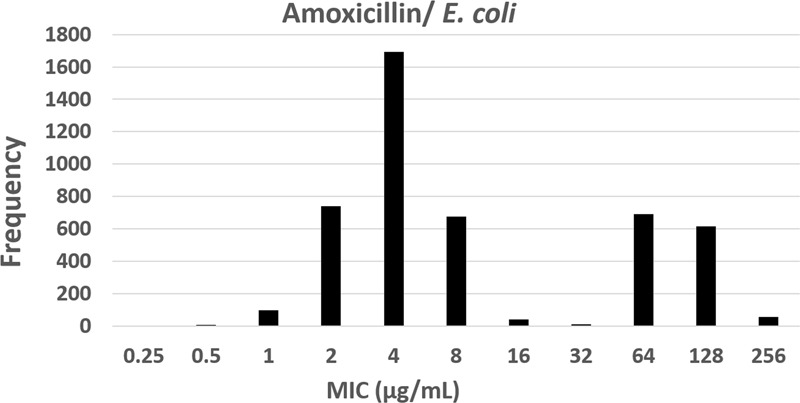
The epidemiological cut-off (ECOFF) of amoxicillin for *Escherichia coli*; ECOFF is the MIC (8 μg/mL) that best separates the two sub-populations of the observed MICs (from 0.25 to 256 μg/mL) distribution, i.e., the wild-type population and of the non-wild-type population. ECOFF is a *parameter* that can be determined using simple visual inspection in the case of a clear bimodal distribution (as here) or by statistical techniques when greater certainty of the estimation is required (EUCAST raw data).

For a given microbial species and agent, the ECOFF is the highest MIC for organisms devoid of *phenotypically detectable* acquired resistance mechanisms. It defines the upper end of the wild-type MIC distribution (**Figure 3**). ECOFFs allow detection of resistance to AMDs as a biological phenomenon. This may or may not be clinically significant but may nevertheless constitute an early warning of acquired resistance. ECOFFs are useful in situations where CBPs have not yet been defined, as with some topical or local agents (e.g., intra-mammary or gastro-intestinal products) or where very different breakpoints may be appropriate for different animal breeds within a given species, preventing the selection of a single CBP.

Epidemiological cut-offs for a given bacterial species are not affected by sampling time, source (human, animal, and environmental) and geographical origin and are therefore biological *parameters*. A CBP can never be lower than the ECOFF (see rule 10.2 of the SOP 1.2 (European Committee on Antimicrobial Susceptibility Testing, 2013) to prevent artificially classifying part of the wild-type population as being resistant.

In order to define an ECOFF, MIC distributions for a given bacterial species from different epidemiological and clinical sources are combined. The proportion of wild-type relative to non-wild-type may change but the boundary separating them does not. The criteria for MIC distribution data sets acceptable for ECOFF estimation are defined by EUCAST. In summary, a minimal total number of 100 MIC values in the putative wild-type population for each bacterial species, originating from at least five accepted MIC data sets, are required to define an ECOFF.

Epidemiological cut-off values are not only used for CPB determination but also for surveillance programs, when detection of acquired resistance mechanisms is relevant.

The numbers of MIC distributions for veterinary pathogens are increasing and are available on the EUCAST website. VetCAST presently has a library of more than 25,000 individual MIC results. Stakeholders are encouraged to send their in-house data for aggregation, after review by VetCAST, with the already published MICs [for details see (Kahlmeter, 2014)].

## The PK/PD Breakpoint

**FIGURE 4:**
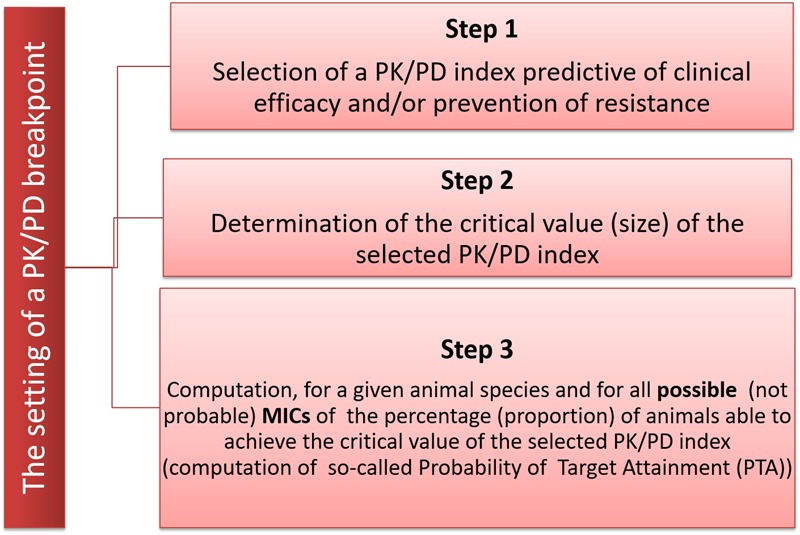
The three steps for the determination of a PK/PD breakpoint. The first step is to select one of the two PK/PD indices predictive of clinical efficacy, i.e., either the time for which plasma concentration remains above the MIC during the dosage interval (*f*T > MIC) or the ratio of area Under the plasma Concentration curve over the MIC (*f*AUC/MIC); the second step is to determine the size of the selected index required to ensure clinical and bacteriological efficacy. The third step is to determine, using Monte Carlo Simulation, the highest possible MIC for which a given percentage of animals in the target population (e.g., a prediction interval of 95%) is able to achieve the selected PK/PD index.

In humans, the PK/PD breakpoint set by EUCAST is generally taken as the highest MIC for which a selected PK/PD index can be achieved in the target population, given the standard dosing regimes and taking into account the lower 95–99% prediction intervals for the population (Mouton et al., 2012). For veterinary medicine in the EU, reference to PK/PD concepts was first introduced in 2016 by EMA/CVMP in its latest guidance on the demonstration of efficacy of antimicrobial substances, although only for pre-clinical investigation (European Medicines Agency, 2016). Currently, no robust clinical data exist for veterinary medicinal products to support any PK/PD breakpoint. Because of this, a PK/PD breakpoint for veterinary medicine can only be established after exploring a range of possible (not probable) MICs, within which a clinical cure can be expected from application of generic pre-clinical and clinical PK/PD principles. In others words, the PK/PD breakpoint, as understood by EUCAST, will be rather viewed for VetCAST as a PK/PD cut-off, i.e., VetCAST will compute a series of Probability of Target Attainments or PTA (*vide infra* for explanation of PTA) from plasma concentration profiles and with no consideration of clinical data. This is also the same procedure adopted by CLSI/VAST under the name of PD_CO_ (Clinical and Laboratory Standards Institute, 2008). The range of MICs explored will not be restricted to wild-type organisms, as a CBP can be greater than an ECOFF with the currently recommended dosage regimen. The PK/PD breakpoints may vary with pathogen group, and both PK data and corresponding possible MIC values are required to compute PK/PD breakpoints. In veterinary medicine, some diseases can be associated with several veterinary pathogens; examples are bovine respiratory disease complex, swine respiratory disease etc. When several causative agents are involved, that causative agent with the highest possible MIC values will be used to compute the PK/PD breakpoint (**Figure 4**). The following are required to establish the PK/PD breakpoint: (i) the PK disposition of the drug in the target animal species following a specific route of administration, (ii) the target value of a selected PK/PD index for bacterial pathogens of relevance predicting a high likelihood of clinical/bacteriological cure, and (iii) the usual range of MICs for a given pathogen for a specific disease.

The PK/PD index selected to compute a PK/PD breakpoint can be viewed as a surrogate of efficacy. Practically, the clinical efficacy of AMDs can be correlated with one of the two following PK/PD indices:

• The percentage of time that plasma concentration of unbound drug remains above the MIC during the dosage interval (*f%*T > MIC); this index is typically that seen with beta-lactams, and is expressed as a percentage of the dosage interval in the steady-state condition.
• The ratio of the Area Under the Plasma Concentration-time curve for free drug to MIC (*f*AUC/MIC); this index is seen with aminoglycosides, fluoroquinolones, macrolides and tetracyclines.
• It should be noted that the ratio of the maximum free plasma concentration and MIC, *f*C_max_/MIC was historically seen with aminoglycosides but is now losing favor.
• The italic *f* indicates that these PK/PD indices should be related to the free (unbound to plasma protein) AMD plasma concentration, because only the free AMD has antibacterial activity. Many reviews (Hyatt et al., 1995; Ambrose et al., 2007; Craig, 2014b) have explained the origin and usefulness of these indices, including their application in veterinary medicine (Toutain, 2002; Toutain et al., 2002; McKellar et al., 2004; Lees et al., 2006; Papich, 2014; Ahmad et al., 2016).

After selecting the index appropriate to the AMD, the numerical target value to be achieved under steady-state conditions (for multiple dose administrations) to predict clinical efficacy must be established. In veterinary medicine, this has historically been done either by using an experimental *in vitro* system (e.g., time-kill curves) or *in vivo* studies in the target species. However, the availability of *in vivo* data for this purpose is rare. The determination of the target value of the PK/PD index for valnemulin in poultry using an intratracheal *Mycoplasma gallisepticum* infection model (Xiao et al., 2015) is an example. When there are no specific veterinary data, a default value of the predictive index is selected from human medicine. The rationale for using values from human medicine is that they are frequently derived from studies conducted in animal disease models (Craig, 2014a).

Target values of these indices indicate the systemic (*in vivo*) exposure, normalized by MIC, required against each pathogenic bacterial species; the target value itself does not depend on animal characteristics, thus conferring on them a generic validity across animal species. PK data are required only to define the specific doses required to achieve the targeted index value. For example, for beta-lactams, a typical target value for *f*T > MIC of 25–40% of the dosage interval for Gram-positive pathogens and 40–50% for Gram-negative pathogens is associated with a high likelihood of success both clinically in humans and in rodent models (Craig, 1998).

The final step in the development of PK/PD breakpoints comprises the determination of the percentage of animals, in the treated population, for a particular dosing regimen, likely to attain the target value of the selected PK/PD index, across a range of relevant MIC values. Statistically, the PK/PD breakpoint is related to the notion of a prediction interval (PI). The appropriate choice of percentile (for instance 90, 95, and 99%) has not yet been decided at VetCAST, but VetCAST anticipates that, due to the large inter-animal variability and the paucity of data, a very wide prediction interval could have large consequences for the establishment of a CBP.

The estimation of the PK/PD breakpoint requires firstly the building of a population PK model to quantify *typical* PK parameters and their between-subject variability. This population model is used to generate *in silico*, by Monte Carlo simulation (MCS), a large sample of plasma disposition curves (typically 5000). This virtual *in silico* population is then used to determine the different percentages of animals for which the target PK/PD value will be attained at different possible MICs. These percentages are the Probability of Target Attainment or PTA (also historically termed Target Attainment Rate or TAR). The principles of this stochastic approach have been described in human medicine for AMD (Ambrose and Grasela, 2000; Dudley and Ambrose, 2000; Drusano et al., 2001) and implemented recently in veterinary medicine for tulathromycin (Toutain et al., 2016) and oxytetracycline (Lees et al., 2016) in calves and amoxicillin in calves (Lees et al., 2015) and pigs (Rey et al., 2014).

VetCAST will take into account several issues to derive the PK/PD breakpoint. The first concerns PK data; these may be raw data generated by several stakeholders (mainly the pharmaceutical industry but also academic laboratories) or alternatively published/reported PK parameters (AUC, *C*_max_, Volume of distribution and terminal half-life) as determined for some drugs by VAST/CLSI and by others (Schwarz et al., 2008; Maaland et al., 2013). In absence of raw data, average parameter values and their standard deviation will be considered even if this approach is not ideal. However, VetCAST is aware that accounting for inter-animal variability in veterinary medicine will very likely decrease the numerical value of some PK/PD breakpoints. This is illustrated by the decrease of a PK/PD breakpoint by up to two dilutions for amoxicillin in pigs: using average PK parameters MIC values of ≤0.5 mg/L were considered as “susceptible” for respiratory tract pathogens (Schwarz et al., 2008), whereas (Rey et al., 2014) using MCS reported that a PTA of only 10.5% was achieved at this MIC for an oral dose of 10 mg/kg twice daily (Rey, 2011).

The availability of individual animal PK data from several sources for a given AMD would allow VetCAST to perform a meta-analysis by aggregating raw PK data (see Li et al., 2015 for a review on value of meta-analysis in veterinary medicine) or by statistical aggregation of summary data of PK parameters and their associated variances. This would be based on a VetCAST guideline on pharmacokinetic data handling.

For a given drug, a population PK analysis using a non-linear mixed effect model is the only acceptable way to measure, with equity, i.e., in a well-balanced manner, available concentration data and covariates. This approach allows documenting the various sources of variability of both biological origin (e.g., breed, sex, age, and health status) and non-biological origin (e.g., study design, sampling times, tested doses, analytical techniques and missing data). Most PK data in veterinary medicine are generated pre-clinically in healthy animals. VetCAST anticipates gaining access to information derived from the treated patient population, as the new EMA guidelines may formally encourage veterinary drug companies to perform targeted-population PK investigations during clinical trials (European Medicines Agency, 2016). This will also improve evaluation of the correlation between individual drug plasma concentrations and clinical outcome.

The advantage of population PK is its ability to detect and quantify differences between breeds, formulations or any covariates that might impede the use of a single final CBP for a given AMD and a given pathogen. This may occur when the AMD is marketed under very different formulations or modalities of administration. This applies to AMDs administered both orally (in medicated food or drinking water – collective treatment) and intramuscularly using long-acting, depot formulations that are commonly used in veterinary medicine. Amoxicillin use in pigs provides an example of this difficulty (Rey et al., 2014), and VetCAST will prepare *ad hoc* expert comments to address issues such as a large difference of some dog breeds to other breeds or to the influence of the gastrointestinal physiology (e.g., ruminant vs. pre-ruminant) that may need to be considered in the selection of CBPs.

Another challenging situation, specific to veterinary medicine, is the case of time-dependent AMDs for which *f%*T > MIC is considered as the appropriate PK/PD index. This challenge arises because the target value of this PK/PD index depends on the shape of the plasma exposure curve which may differ widely between the many modalities of AMD administration encountered in veterinary medicine. As an example for oral administration, the possibility of pulse dosing of AMDs by gavage at pre-determined intervals, or alternatively the administration of AMD incorporated in food or drinking water, thus approximating a sustained oral infusion over the day, may affect the possibility to select a single common final CBP. As it is unrealistic to propose several CBPs for a single drug for a given animal species, VetCAST will explore alternatives, in particular the possible use of *f*AUC/MIC as a default index, as it is not influenced by the actual shape of the disposition curve. The objective is to explore the possible similarity of PTA obtained when using AUC/MIC as the PK/PD index but not using T > MIC for the multiple modalities of drug administration and/or for different formulations that are frequently used in veterinary medicine. It is well established that the three PK/PD indices exhibit some co-linearity (Corvaisier et al., 1998); and this has also been reported in veterinary medicine (Greko et al., 2003). More importantly, it has been shown, using a semi-mechanistic *in silico* model, that AUC/MIC is the most appropriate index, when the terminal half-life is relatively long relative to the dosage interval, even for beta-lactams (Nielsen and Friberg, 2013; Kristoffersson et al., 2016). Validation of AUC/MIC as an index of efficacy to ensure not increasing the risk of promoting antimicrobial resistance when T > MIC is a relevant metric, would greatly facilitate the assessment of most long-acting formulations.

The main difficulty for these long-acting formulations is to define their actual duration of efficacy. In human medicine, PK/PD values are obtained under steady-state conditions, because most AMDs are administered on a daily basis using multiple dosing treatment. For this modality of multiple doses, the duration of action is implicitly the same as the duration of treatment. For long-acting formulations, or for drugs with a long terminal half-life, the common practice in veterinary medicine is to administer a single dose. Consequently, estimation of *f*AUC/MIC is complicated by the need to first determine the duration of action of the product, and this information is often lacking in the *Summary of Product Characteristics* dossiers. The solution is to compute PK/PD index values for different incremental time periods with steps of 24 h and to derive a PK/PD breakpoint for each total duration of activity (0–24, 0–48, 0–72 h…). This approach implies a trade-off between the target value of the PK/PD index and the claimed duration of activity, and the final CBP will not only be dose-dependent, as in human medicine, but also, for these types of drugs/formulations, a function of the claimed duration of AMD activity. Therefore, a formulation/substance for which a company wishes to claim a long duration of action will ineluctably have a lower “average 24 h PK/PD index value,” and ultimately a lower CBP. An example of this approach has been recently published for tulathromycin, a macrolide with a terminal half-life of 84 h in calves (Toutain et al., 2016). As explained in the section on how VetCAST will establish the CBPs, ECOFFs will be recommended in situations where a single CBP cannot be firmly established, i.e., a single CBP to cover the multiple modalities of AMD administration of different formulations marketed in the EU.

## The Clinical Cut-Off Value

The third piece of information to establish a CBP should be directly related to clinical outcomes and requires specific investigations during prospective clinical trials. At EUCAST what is established in patients, who yielded bacterial clinical isolates for MIC testing, is the value of the calculated PK/PD index (e.g., AUC_24h_/MIC or T > MIC) that best discriminates between clinical outcomes (e.g., cure vs. non-cure). Currently there is no veterinary clinical example where such an exposure/effect relationship has been demonstrated in a clinical setting. Furthermore, such a relationship cannot be always readily obtained. A retrospective analysis of 16 randomized clinical trials, conducted to explore the relationship between *in vitro* MICs of tilmicosin against *Mannheimia haemolytica* and *Pasteurella multocida* and the outcome of tilmicosin treatment, showed a tendency for greater treatment success against infections with *M. haemolytica* isolates classified as susceptible compared to those categorized as either intermediate or resistant. However, this difference was not statistically significant (*P* = 0.08) (McClary et al., 2011). It is in practice very difficult to distinguish between curable vs. non-curable sub-populations based solely on a MIC, especially when an *ad hoc* trial has not been designed for this purpose. This is because AST only measures the *in vitro* interaction between pathogen and drug. It does not take into consideration disease severity and pathogen load in the biophase, immunological response to the disease, secondary mechanisms of action of AMDs, or the numerous other clinical factors (such as co-medications, timing of treatment initiation relative to development of the disease and individual drug disposition) that may influence treatment outcome (Turnidge and Paterson, 2007; Martinez et al., 2013).

Clinical correlation with AST (MIC), even when it does exist, may be difficult to establish, because most clinical trials do not include enough animals infected with non-wild-type organisms (high MIC). Furthermore, there are often insufficient cases of clinical failure, irrespective of the MIC, because clinical trials are planned to provide evidence of efficacy rather than to quantify lack of efficacy.

Despite these complications, VetCAST will consider clinical data. As EMA requires collection of bacteriological samples during clinical trials, unexplored proprietary databases in veterinary medicine, from which a clinically significant MIC could be reliably determined, are likely to exist. In addition, EMA is now encouraging the use of population PK for veterinary medicine and it can be anticipated that VetCAST would also explore as for human medicine the relationship between some PK/PD index as AUC/MIC and the treatment outcome, which can be either clinical or bacteriological cure. It would be possible to apply classification and regression tree analysis (CART) to identify the clinical cut-off, if appropriate data (AUC/MIC, T > MIC but also AUC/MBC, Dose/MIC…) vs. outcomes are rich.

Irrespective of the clinical predictive variable of clinical outcomes considered for statistical analysis (MIC, AUC/MIC…), clinical trials should be conducted according to acknowledged guidelines (FDA, EMA, and VICH) in order to be considered for such an assessment. Furthermore clinical isolates from these trials should be subjected to a subsequent MIC determination.

## Data Assessment for Local AMD Administration: The Case of Mastitis in Dairy Cattle

A challenging issue for AMD use in veterinary medicine concerns local administration, notably for mastitis treatment in dairy cattle. The potential advantage is that high local drug concentrations and exposures may be achieved at the site of infection with a relatively low dose of AMD, thus minimizing unwanted systemic side-effects and ingestible tissue residues (Gruet et al., 2001). As the milk concentration can be very high and not in parallel with the plasma concentration profile, it is logical to hypothesize that the concentration in milk is the most appropriate for evaluating infection site exposure.

Veterinary-specific CBPs for intra-mammary AMD administration have been established by the CLSI/VAST committee and include a penicillin/novobiocin combination, ceftiofur, pirlimycin, and cefoperazone (Clinical and Laboratory Standards Institute, 2007). CLSI/VAST adopted the straightforward approach of evaluating the time above which the milk concentration is above a critical MIC (T > MIC) with a recommended dosage regimen. For example, for cefoperazone, it was concluded that a concentration of 2 mg/kg milk could be achieved, after regular dosing, for the entire 24 h dosing interval with the 250 mg product and for approximately 80% of the 48 h dosing interval with the 100 mg product (Fessler et al., 2011). Consequently, a MIC breakpoint of 2 mg/L was selected for the category “susceptible” for all bovine mastitis pathogens. However, the udder cannot be simply considered as “a well-stirred milk pot,” and several conceptual, major PK and PD issues will be examined by VetCAST before specific recommendations for CBP determination can be established for this and other local routes of administration. As stated above, ECOFFs will be recommended in situations like this where CBPs cannot be firmly established.

## The Establishment of Clinical Breakpoints by the VetCAST Steering Committee

The CBP will be determined by the VetCAST steering committee after completion of the scientific assessment. As explained above, members of this group will not be representative of any national breakpoint committee but are acknowledged as *intuitu personae* experts. As stated by Turnidge and Paterson (2007) it is essential that the membership of any breakpoint-setting committee includes persons to encompass a range of skills; and this will include for VetCAST scientists in the fields of clinical microbiology, epidemiology, infectious diseases, pharmacology, clinicians and regulatory affairs. Declarations, confirming the absence of any conflict of interest, for those involved in any step of the establishment of a CBP will be updated annually. At VetCAST, decision taking will be carried out according to the EUCAST procedure through a consensus process. The principal reason for adopting consensus is that EUCAST is an EU consensus-driven organization.

Operationally, CBPs will be established by VetCAST from the MIC cut-off values derived from (i) the ECOFF (ii) the PK/PD breakpoint and when available (iii) the clinical cut-off. Alternative to the latter is to consider some metrics predictive of clinical outcome (Dose/MIC, AUC/MIC…) as computed during the scientific assessment step. There is no generally accepted formula or mechanism for combining these different pieces of information into a single CBP to define the *Susceptible* category. Decisions in the final analysis are a matter of judgment by experts drawn from several disciplines and based on reflective consideration of all available information.

Historically, most emphasis in veterinary medicine has been given to epidemiological and clinical considerations and for CLSI/VAST, the so-called PK/PD cut-off (named CO_PD_) is a relatively recent innovation. PK/PD cut-off differs from PK/PD breakpoint as understood by EUCAST by the fact that a PK/PD cut-off is derived only from PK data without clinical considerations. It is the VetCAST view that selecting a MIC having a robust clinical meaning is challenging for a range of reasons explained above. Therefore, VetCAST supports the conclusion of Turnidge and Paterson (2007) that much of the pivotal information is embedded in the PK/PD breakpoint. This is because the PK/PD breakpoint is a hybrid value, incorporating all three principal components (microbiological, pharmacological, and clinical) predicting clinical efficacy. Operationally, if the PK/PD breakpoint is below the ECOFF, it probably means that the current dosage regimen for that AMD is too low to treat the wild-type population. In this case, VetCAST will not establish a CBP dividing the wild-type MIC distributions (Arendrup et al., 2009). However, it should be emphasized that VetCAST has no authority, at that level, to recommend a change of dosage regimen. On the other hand, a CBP can be established if the PK/PD breakpoint is greater than or equal to the ECOFF, and, under these circumstances, clinical data, when available should be considered to support the PK/PD breakpoint.

## VetCAST Communication and Consultation

For VetCAST, dissemination of information, concerning standards, guidelines, SOPs, ECOFF, PK/PD breakpoint, CBPs and any additional issues relating to susceptibility testing, will be interactive with stakeholders. The latter include international and national agencies, professional organizations, other committees such as CLSI/VAST, prescribers, scientific news media, and other interested groups. The VetCAST steering committee will propose a tentative CBP based on data assessment for public consultation. Stakeholders such as pharmaceutical companies or any organizations may subsequently send comments to or request meetings with the VetCAST Steering Committee. This open communication will facilitate a possibility to take new data into consideration, to provide further discussion or to seek clarification of the VetCAST rationales for determining the CBP.

In terms of communication, a crucial issue will be to explain and communicate the fact that new or revised VetCAST CBPs or ECOFFs can have many repercussions for microbiological laboratories, similar to those reported by a focus group regarding CLSI policy (Jenkins and Jerris, 2011). This includes the development and validation of new laboratory methods and, more importantly, the possibility of reporting as susceptible those isolates that would be classified as resistant, based on the previously employed breakpoint (or ECOFF), or conversely reporting as resistant isolates previously classified as susceptible when the CBP is decreased (see also Hombach et al., 2012; Heil and Johnson, 2016). For similar reasons, the prevalence of resistance, assessed from aggregated AST results at regional, national or international levels, might be substantially altered, especially when a CBP, rather than ECOFF, is used to report resistance.

The independent status of VetCAST, when it expresses general views to all groups concerned with AMD usage and dosage, is crucial.

VetCAST will communicate via the EUCAST website and regularly consult with stakeholders. All relevant material will be freely available to any testing laboratory, including those in resource-poor settings, to provide an updated and readily available reference for interpreting pathogen susceptibilities to AMDs.

## Expert Comments for Stakeholders

An important item of communication, after establishing a CBP, is to promulgate a set of expert comments and/or recommendations associated with the new VetCAST CBP. Expert rules are intended to assist clinical microbiologists in the routine interpretation of AST data and to suggest the most appropriate actions to be taken in response to specific AST results, i.e., not to be restricted to the simple reporting of *S, I*, or *R*. As for EUCAST, expert rules (Leclercq et al., 2013) for VetCAST will include recommendations on reporting (including the suppression of results when possibly misleading) and editing of results on the basis of an inferred resistance mechanism, inferring susceptibility to other agents from reported results etc. For veterinary medicine, a single CBP for each AMD is unlikely to cover all clinical situations and other circumstances (local vs. systemic administration, curative vs. metaphylaxis dosing, possible effect of breed, formulations…). Therefore, these expert rules will place data in perspective, based on microbiological and clinical considerations, with warnings on the interpretation, relevance and limitations of the VetCAST CBP. Further, they will advise on additional tests that might be required. These expert rules will be regularly updated and could be incorporated into the software of susceptibility testing devices.

## Passive Resistance Surveillance and CBPS

As indicated above, AST results can be aggregated at different geographical levels within Europe, similar to that of the database of the European Antimicrobial Resistance Surveillance Network (EARS-Net). Passive surveillance is less expensive than other surveillance strategies, and is the most effective approach for detecting rare and potentially emerging resistance (Mather et al., 2016).

For surveillance purposes especially if CBPs have not been harmonized, the most relevant criterion is the ECOFF, which separates the wild-type from the non-wild-type population, and therefore does not differ between countries or over time. Therefore, one of the priorities of VetCAST will be to collect MICs of many isolates of interest to both animal and public health, in order to establish science-based ECOFFs. This goal could be shared with CLSI/VAST with the joint promotion of a set of international ECOFFs.

## Conclusion

To ensure the dominant role of AST as a phenotypic diagnostic test for veterinary medicine, it is crucial to improve its accuracy and predictive clinical value. VetCAST aims to do this by definition of science-based interpretive criteria and CBPs based on integration of microbiological *in vitro* potency with *in vivo* pharmacological and clinical data as described in this position paper. As part of this process, VetCAST will address complexing issues that are specific for veterinary medicine, e.g., long-acting formulations, in feed medication, frequent use of topical formulations, and animal species and breed-specific differences in pharmacokinetic drug profiles.

## Author Contributions

P-LT drafted the paper. All the co-authors critically reviewed its several drafts.

## Conflict of Interest Statement

The authors declare that the research was conducted in the absence of any commercial or financial relationships that could be construed as a potential conflict of interest.

## Footnotes

1 http://www.eucast.org
